# Epistemic Blame and the Normativity of Evidence

**DOI:** 10.1007/s10670-021-00430-9

**Published:** 2021-06-14

**Authors:** Sebastian Schmidt

**Affiliations:** Philosophisches Seminar, University of Zurich (UZH), Room G-228, Zürichbergstrasse 43, 8044 Zurich, Switzerland

## Abstract

The normative force of evidence can seem puzzling. It seems that having conclusive evidence for a proposition does not, by itself, make it true that one ought to believe the proposition. But spelling out the condition that evidence must meet in order to provide us with genuine normative reasons for belief seems to lead us into a dilemma: the condition either fails to explain the normative *significance* of epistemic reasons or it renders the *content* of epistemic norms practical. The first aim of this paper is to spell out this challenge for the normativity of evidence. I argue that the challenge rests on a plausible assumption about the conceptual connection between normative reasons and blameworthiness. The second aim of the paper is to show how we can meet the challenge by spelling out a concept of epistemic blameworthiness. Drawing on recent accounts of doxastic responsibility and epistemic blame, I suggest that the normativity of evidence is revealed in our practice of suspending epistemic trust in response to impaired epistemic relationships. Recognizing suspension of trust as a form of epistemic blame allows us to make sense of a *purely* epistemic kind of normativity the existence of which has recently been called into doubt by certain versions of pragmatism and instrumentalism.

## Introduction

Do epistemic norms provide us with normative reasons for compliance? Such norms tell us, very roughly, that we should believe what we have sufficient evidence for, and that we should refrain from believing what we lack sufficient evidence for. Recently, epistemologists have questioned that epistemic norms have genuine normative significance. Susanna Rinard, for instance, argues “that only pragmatic considerations are genuine reasons for belief. That is, purely evidential considerations—evidential considerations that are not also pragmatic reasons—do not constitute reasons for belief” (2015, 219). To illustrate this view, consider a case in which you happen to come across the newest celebrity gossip in a magazine that you know to be reliable. Assume that you know that having a true belief about the gossip is not, nor will ever be, of any practical value for you, and that you are not curious about such gossip at all. Now consider the following questions:

- *Should* you believe the gossip?
- Are you *blameworthy* or *criticizable* if you fail to believe the gossip?

Or consider a slightly altered case in which you happen to *believe* some celebrity gossip although you are aware that the magazine in which you came across it is *not* reliable. Your belief is not based on sufficient evidence. We can again stipulate that you know that nothing bad will ever come from having such an ill-based belief about this unimportant matter that is of no interest to you. Now consider the following question:

- Are you *blameworthy* or *criticizable* for having this belief?

The view under consideration in this paper would reply with “no” to all these questions. Let us call this view “anti-normativism about evidence” (short: ANE). Proponents of ANE argue that mere evidence does never provide us, *by itself*, with a (normative) reason for belief. Next to Rinard’s view, recent instrumentalists about reasons for belief commit to ANE. Asbjørn Steglich-Petersen and Mattias Skipper argue “that evidence for *p* speaks in favor of believing *p* only in context where there is a practical reason to pursue the aim of coming to a true belief as to whether *p*” (2019, 9), and that therefore “it is strictly speaking false to say that evidence *by itself* constitutes a normative reason for belief” (2020, 114). Similarly, Maguire and Woods (2020) have recently denied that purely epistemic norms provide us with reasons.^1^ They compare epistemic norms with rules of games: we only have a reason to comply with each if we have a practical (prudential or moral) reason to engage in the relevant practice. That is, I have a reason to move a chess piece according to the rules only if I have a practical reason to play chess; analogously, they argue that I have a reason to believe that p only if I have a practical reason to play what Maguire and Woods call “the game of belief”.^2^

The normativity of evidence is at stake in contemporary epistemology. What could possibly count as a satisfying reply to ANE? This paper is devoted to answering this question. I argue that, in order to get clear about the normative significance of evidence, we need to think about the reactive attitudes that are appropriate towards violations of purely epistemic norms. I reach this claim by first spelling out the dilemma of explaining why epistemic norms matter without thereby rendering them practical (Sect. 2). This dilemma will allow us to pin down a challenge for normativism about evidence: finding a satisfying conception of a distinctively epistemic kind of blame (Sect. 3). I then propose, in outline, a reply to this challenge (Sect. 4). I suggest that we hold each other answerable to epistemic norms by showing reactive attitudes towards each other’s epistemic failures—mainly suspension of epistemic trust (cf. Boult, 2020, 2021). The normativity of evidence can become intelligible by understanding this practice. For the reactive attitudes within our epistemic practice reveal the normative significance of purely evidential considerations.

The result of the paper is that we should not submit too quickly to treating the epistemic as hostage to the practical. Rather, we should first engage in the project of understanding the distinctive normative significance of purely evidential considerations by appealing to our responsibility for (non-)compliance with epistemic norms. Only if it were to turn out that this project fails—because, say, it turns out that there is no distinctive responsibility attached to the purely epistemic—we would be justified to endorse ANE. However, proponents of ANE have not yet provided arguments that this project fails. Doing so would require them to engage with recent accounts of epistemic blame and responsibility for belief. The paper thus shifts the dialectical burden to proponents of ANE and connects debates within contemporary epistemology.

## The Challenge for the Normativity of Evidence

This section argues that normativism about evidence—the view that purely evidential considerations provide us with reasons for belief—faces a dilemma. I first outline two strategies for finding a plausible content of epistemic norms (Sect. 2.1). I argue that both strategies give rise to the same dilemma for normativism (Sects. 2.2). This will then allow us to formulate the central challenge for normativism in Sect. 3, and thus to see the intuitive appeal of ANE.

### Replying to the Clutter-Objection: Background Conditions on Epistemic Norms

Consider the following rough first approximation towards formulating an epistemic norm (also mentioned at the beginning of this paper):

(EN) One ought to believe everything that is sufficiently supported by one’s evidence.

Gilbert Harman (1986, 12) points out that (EN) implies that we should clutter our minds with uninteresting implications of our beliefs. My current belief-stock implies the proposition that [I am sitting in my office *or* the moon is made of cheese *or* there is no Corona virus *or* there is no human-induced climate change *or* …]. This disjunctive proposition is true right now while I am sitting in my office. It is true because the first claim, that I am sitting in my office, is true. At the same time, it seems that I do not always believe this disjunctive proposition while I am sitting in my office. Most importantly, it seems that I would not be *blameworthy* or *criticizable* in any sense for *not* believing such disjunctive propositions. It follows, so it seems, that there is no unconditional norm to believe everything that is sufficiently supported by my evidence.^3^

In reaction to this, we might modify the epistemic standard so that its violation more plausibly gives rise to serious criticism. We might propose background conditions for *when* we are required to believe what our evidence sufficiently supports. These background conditions should fulfill two criteria:

- They must make it plausible that the subject is, at least normally or in paradigm cases of an epistemic norm violation, blameworthy or criticizable for not complying with the epistemic norm when the background conditions are fulfilled.
- They should not render the norm *practical* rather than *epistemic*.

﻿Call (a) the criterion of *significance*, and (b) the criterion of *content*. (b) makes sense as a criterion on epistemic norms for our purposes because the aim of the normativist is to preserve a purely epistemic kind of normativity. But why (a)?

The guiding idea behind (a) is that the significance of a norm expresses itself in the reactive attitudes that we show towards violations of the norm. For instance, the significance of a moral requirement will make it often—in absence of an excuse or exemption—appropriate to show resentment or indignation. These emotions are expressions of the normative significance we attach to the moral requirement because they are appropriate in face of its violation. Similarly, if there are distinctively epistemic norms that provide us with reasons for compliance, then we should expect there to be distinctively epistemic reactive attitudes that we show towards the violation of those epistemic norms. In this vein, Antti Kauppinen understands genuine norms (as contrasted with mere evaluative standards) as “rules that someone is accountable for conforming to in suitable circumstances” (2018, 3).

Here is an argument for (a). *Why* is it false that we should clutter our minds with all the implications of our beliefs? If we would accept that epistemic norms require us to clutter our minds, then we would constantly violate an epistemic norm by not drawing all the implications from our beliefs. However, this constant violation would have no further significance: we would not normally be blameworthy or criticizable for failing to believe what we epistemically ought to believe. The problem with this is that the normative force of this “ought” would then be mysterious: why comply with this norm if one cannot hold us legitimately responsible for non-compliance? The norm would at best have the force of the norms of etiquette or the rules of a game: we can intelligibly ask *why* we have a reason to comply with the norms of etiquette or rules of a game in a given situation. Such norms do not, by themselves, provide us with reasons. Thus, the best explanation of the intuitive appeal of Harman’s clutter-objection when it comes to trivial implications of our beliefs is that we assume that epistemic norms with normative significance would fulfill (a).^4^

I will return to the connection between epistemic reasons and epistemic blameworthiness in Sect. 3. For now, consider another strategy for finding a plausible norm of belief that is purely epistemic. Instead of proposing background conditions to (EN), we might rather argue that (EN) is not a central epistemic norm at all. In response to Harman’s clutter-objection, we might argue that, although we are never blameworthy merely for failing to believe what our evidence sufficiently supports, there are *other* epistemic requirements that are purely evidential. Specifically, we might defend the following epistemic norm:

(EN*) One ought not [to believe what is not sufficiently supported by one’s evidence.]

(EN*) is not confronted with Harman’s clutter objection: rather than requiring us to believe plenty of propositions we intuitively have no reason to believe, (EN*) merely *prohibits* us to have certain beliefs. Steglich-Petersen (2018) accepts (EN*) but denies (EN): he thinks that evidence alone determines the *permissibility* of belief (which beliefs I am epistemically allowed to have), but he argues that evidence alone never gives us, as he puts it, “positive reason” to believe a certain proposition. Epistemic norms, on this picture, determine the space of doxastic permissibility, but they never require a specific belief—rather, they merely prohibit certain beliefs (cf. also Whiting, 2010, 2013).

However, appealing to a norm of permissibility like (EN*) instead of (EN) won’t help to defend the normativity of evidence against the challenge I spell out in this paper. First, it seems that (EN*) should not be any more plausible to skeptics about the normativity of evidence than (EN). The norm that we ought *to believe everything* that is supported by our evidence faces the problem that it requires us to needlessly clutter our minds. The norm that we ought *not to believe anything* that is *not* sufficiently supported by our evidence faces the reverse problem: it would require us not to have a lot of beliefs we, it seems, have no reason to give up. For example, why should I give up evidentially unsupported but beneficial beliefs? We often overestimate our own abilities or the virtues of our significant others. Arguably, this can promote our self-esteem (cf. Kelly, 2003) or our relationships (cf. Stroud, 2006). It seems that such beliefs are blameless as well. A general norm not to believe what is insufficiently supported by one’s evidence seems *too exclusive*. And a norm to believe anything that is sufficiently supported by one's evidence seems *too inclusive*.

Furthermore, cases of trivial belief pose the same problem for (EN*) as they pose for (EN). What if you believe, in absence of sufficient evidence, that the celebrity gossip in this unreliable magazine is true? Why should it make sense for anyone to blame or criticize you for this belief, if we stipulate that your trivial belief will have no bad consequences? Such trivial propositions seem to pose a challenge to (EN*) as they do to (EN)—as I will illustrate in some more detail in Sect. 2.2 below.

One final clarificatory remark: I will call any form of blame that arises from the violation of a *purely* epistemic norm—i.e., an evidential norm that does not mention any practical considerations—*epistemic blame*. That is, epistemic blame, if there is such a thing, is a kind of negative reaction that is appropriate towards violations of purely epistemic norms like (EN) or (EN*), given suitable non-pragmatic background conditions. I now turn to the idea of background conditions on epistemic norms in some more detail to spell out a dilemma for normativism about evidence that will give rise to a challenge for normativism.

### Proposing Background Conditions: A Dilemma

One way of developing a background condition on (EN) that might allow us to preserve the normativity of evidence is presented by Kiesewetter (2017, 184–5). He responds to Harman’s clutter-objection by proposing that the central standard of theoretical rationality is to believe what one’s evidence sufficiently supports *if one attends to this evidence*. According to this proposal, if I attend to the fact that I have sufficient evidence for a specific disjunctive proposition, then I would be criticizable (because irrational) if I do not come to believe it. Thus, Kiesewetter concludes, there is a sense in which I *ought* to believe it as soon as I *attend* to my sufficient evidence. Analogously, we could propose a background condition on (EN*) by saying that if we attend to the fact that we lack sufficient evidence for p, we ought not to believe p: we would be criticizable if we were to believe p; but we wouldn’t be criticizable for this if we never noticed how our belief lacks evidential support—we wouldn’t count as irrational.^5^

It seems that Kiesewetter’s background condition, while doing a good job in fulfilling criterion (b), does not fulfill (a). There are cases where we attend to sufficient evidence but where it would not make much sense to regard us as blameworthy or criticizable if we, for whatever reason, do not believe what the evidence supports. Take, again, the case in which I come across the newest celebrity gossip in a magazine that I know to be reliable, but I fail to believe the gossip. Assume again that having a belief about the matter is of no importance and that I do not care about whether the gossip is true. Proponents of ANE will argue that there is then no sense in which I am blameworthy, and that it is false that I ought to believe the gossip.^6^

It thus seems that if it does not *matter* whether we believe an evidentially well-supported proposition, it is false that we *ought* to believe it. However, if we instead propose a background condition on sufficient evidence that implies that it always matters whether we comply with the epistemic norm, we seem to end up violating criterion (b): if the norm is only in place when it matters whether we comply with it, then, so it seems, the norm is no longer a purely epistemic norm. It thus seems that there is no background condition on purely epistemic norms like (EN) and (EN*) that fulfills both (a) and (b). The normativist is in a dilemma.

Let us provide this dilemma with additional support by considering a background condition that does not fulfill criterion (b). Steglich-Petersen (2011), after discussing a case of a trivial belief that the subject is not required to have although it is well-supported by the subject’s evidence (23), presents the following partial analysis of reasons for belief:

Necessarily, if S has all-things-considered reason to form a belief about *p*, then [if S has epistemic reason to believe that *p*, S ought to believe that *p*] (24).

Here “epistemic reason” can be read as “sufficient evidence for p”. The conditional then states that *if one has an all-things-considered reason to form a belief about p*, one ought to believe what one’s evidence sufficiently supports. The italicized if-clause is Steglich-Petersen’s proposed background condition for the epistemic standard (EN). Steglich-Petersen could analogously propose a background condition on (EN*): *if one has an all-things-considered reason to form a belief about p*, then if p is *not* sufficiently supported by one’s evidence, one ought not to believe p.^7^

Steglich-Petersen’s “all-things-considered reason to form a belief about p” can, for instance, be a reason for an action prior to the belief.^8^ “Forming a belief about p” might refer to the action of *thinking about whether p*: I may have more or less reason to think about whether something is true. I have some reason to think about whether there will be nice weather during the next days, but I have no reason at all to think about the newest celebrity gossip (I might even have reason to avoid such thinking). Thus, according to one plausible reading, the truth of “S ought to believe that p” in Steglich-Petersen’s analysis is conditional on a practical reason for an action. It says that if we have a reason to bring it about or to maintain that we have a (true)^9^ belief about p, and there is sufficient evidence for p, then we ought to believe that p.

Since we only have a reason to bring a belief about when it *matters* whether we have this belief, Steglich-Petersen’s proposal does a good job fulfilling our criterion of *significance*. Yet his proposed background condition, and thus the proposed epistemic norm, is no longer *epistemic*, because it includes a practical reason (for an action). His proposal thus fails to fulfill our criterion of *content*.^10^

Thus, while proposing non-pragmatic background conditions on epistemic norms (á la Kiesewetter) apparently does not result in doxastic norms that fulfill the criterion of *significance*, proposing a pragmatic background condition (á la Steglich-Petersen) results in doxastic norms that do not fulfill the criterion of *content*. If the normativist about evidence accepts the criterion of significance for epistemic norms, they have to defend the claim that compliance with epistemic norms matters (in a sense) even if we do not equip these norms with a pragmatic background condition. *Prima facie*, it is hard to see how purely epistemic norms could matter by themselves. Therefore, any normativist will, it seems, end up in either of two horns:

(i) Epistemic norms are purely epistemic, but they fail to be significant.
(ii) Epistemic norms are significant, but they fail to be purely epistemic.

We might be tempted conclude from the dilemma that ANE is true: we might think that we should just reject the idea that we are ever *epistemically* blameworthy, and that there is any such thing as a purely *epistemic* kind of normativity.

I think this dilemma points to a serious challenge for the normativity of evidence. However, I will ultimately propose that purely evidential considerations *have* a kind of normative significance: (non-)compliance with purely epistemic norms matters. This requires me to make sense of a notion of epistemic blame. For the significance of a norm expresses itself in our reactive attitudes towards violations of the norm (cf. Sect. 2.1). Thus, giving a satisfying reply to ANE requires us to reconsider the concept of blameworthiness and responsibility for beliefs. For now, however, we should accept that there is a good case to be made for ANE. I now turn to the argument that is the core of this challenge for the normativity of evidence.

## An Argument for Anti-normativism About Evidence

This section presents an argument that constitutes the core of the challenge for normativists (i.e., opponents of ANE) (Sect. 3.1), and it defends one of the two premises of this argument (Sect. 3.2). Replying to the challenge will thus require rejecting the other premise of the argument, which states that there is no distinctively *epistemic* kind of blame. I will turn to the rejection of this premise in Sect. 4.

### The Argument from Doxastic Blameworthiness

Given the dilemma spelled out in Sect. 2, it is now easy to see how the denial of epistemic blame can give rise to an argument for ANE. Take “purely epistemic norms” to refer to (EN) or (EN*), and, if you want to, include any background conditions on purely epistemic norms that do not render the norm practical (like Kiesewetter’s attending condition):

1. Evidence provides us with epistemic reasons for belief only if we can be blameworthy (or criticizable)^11^ for violating purely epistemic norms.
2. We cannot be blameworthy (or criticizable) for violating purely epistemic norms.
3. Thus, evidence does not provide us with epistemic reasons for belief.

According to the view expressed in (3), evidence provides us with reasons only if there is some practical value realized in following the evidence. Even if evidence does not *by itself* provide us with reasons for belief, it *appears* as if it provides us with such reason, because we normally have reason to follow the evidence—but this reason is only instrumental because it derives from our reason for the aim of gaining *practically valuable* true beliefs (cf. Rinard, 2015, 219). ANE just is this practical-instrumental approach to purely epistemic normativity.^12^

Premise (1) has a high *prima facie* plausibility. For it states a very minimal conceptual connection between reasons for belief and blameworthiness. The consequens of (1) states that it must be *possible* to be blameworthy for non-compliance with purely epistemic norms (under adequate non-pragmatic background conditions). (1) states that if this is not possible, evidence does not, by itself, provide us with reasons for belief. That is, (1) states that epistemic blame, as defined at the end of Sect. 2.1, must be conceptually possible if purely evidential considerations are to provide us with reasons for belief.

Remember that, as I have argued in Sect. 2.1, Harman’s clutter-objection gets its grip on us only because we implicitly assume a connection between reasons and blameworthiness. Epistemologists propose background conditions on epistemic norms, like Kiesewetter’s attending-condition or Steglich-Petersen’s reason for forming belief about whether p, precisely because they want to make sense of the *significance* of these norms: they want to explain why it *matters* to us whether we comply with the norms—why we can be blamed or criticized if we fail to comply with them. The point of spelling out a notion of epistemic blame is to understand the normative force of evidence: why it matters to comply with epistemic norms.^13^

Yet most importantly, even if we were to reject that we need to be *always* blameworthy or criticizable for failing to comply with purely epistemic norms if evidence is to provide us with epistemic reasons for belief, this would not refute (1). For according to (1), it must merely be *possible* to be blameworthy for such non-compliance: there must be some possible cases in which we are blameworthy in virtue of the fact that we violate purely evidential norms in order for evidence to provide us with epistemic reasons for belief.

If (1) is indeed such a weak claim, then we should expect (2) to be more controversial to allow for the controversial conclusion ANE. Yet we saw that there is a strong *prima facie* case for (2) to be made by appealing to cases of trivial belief. It is not straightforward in what sense a person who violates a purely epistemic norm is blameworthy when nothing of practical value hinges on whether the person complies with the norm. That we *can* be blameworthy for violating a purely epistemic norm—i.e., non-(2)—seems to imply that we sometimes *are* blameworthy in cases of trivial belief. For cases of trivial belief are the cases in which we have isolated any non-epistemic factors—like the factor that there is a practical reason to consider our evidence carefully, or the factor that that it would be morally good to believe what one’s evidence supports, or that having a certain belief would be disrespectful to a person. If there is never any blameworthiness left after we have isolated these non-epistemic factors—as it seems to be the case in cases of trivial belief—then it seems that there is no such thing as a distinctively epistemic kind of blameworthiness, and thus, given (1), no purely epistemic normativity. (I return to how normativists about evidence can deal with cases of trivial belief in Sect. 4.3.)

I will first defend (1) against objections. I thereby show that a challenge for normativism about evidence consists in arguing against (2)—i.e., in finding a good account of the normative significance of evidence by appealing to the concept of epistemic blameworthiness.

### Some Worries About Blameworthiness as a Precondition on Reasons

The first objection to premise (1) points out that children or some non-human animals can act for reasons but cannot be blameworthy if they fail to do what is decisively supported by their reasons, because they are not responsible agents. Analogously, some children and animals might be considered has having reasons for belief even though they cannot be blameworthy for violating purely epistemic norms. Thus, (1) does not seem to hold.

The objection can easily be met by pointing out that (1) does not imply that *everyone* can be blameworthy for violating purely epistemic norms. As I have explained in the last subsection, (1) merely states that it must be possible to be blameworthy in a distinctively epistemic sense if evidence is to provide us with epistemic reasons for belief. If there are some beings who are not fully responsible agents or have not yet developed to fully responsible agents, they might also be exempted from epistemic blame. But that does not count against the idea that those beings who are fully responsible for their actions and beliefs must sometimes be subject to epistemic blame if evidence is to provide us with epistemic reasons.

Secondly, one might wish to deny (1) if one is an objectivist about the meaning of “ought” and “reasons”. Objectivists deny a close connection between failing to do what one ought to and being blameworthy (a connection usually utilized or argued for by subjectivists).^14^ Objectivism about practical reasons states that “S ought to φ” means that φing is the best option, no matter whether S is in a position to know or has some kind of cognitive access to the fact that φing is the best option. For example, if my house is burning even though I do not have any clue that it is burning, the objectivist would claim that I ought to leave the house. The subjectivist would deny this and say that I only *ought* to leave the house if I—in some way or another—have cognitive access to the fact that the house is burning. If I have access to that fact but do not leave the house, then I am blameworthy. According to the objectivist, it could be the case that I am not blameworthy, and yet it is true that I *ought* to leave the house (namely, if I am not in a position to know that it is burning). It thus seems that, if we are objectivists, we do not think that there is any close connection between what we ought to do or what we have reason to do, on the one hand, and blameworthiness, on the other. This suggests that, *prima facie*, adopting an objectivist theory about reasons for belief would pose a problem to (1).

However, (1) is uncontroversial for objectivists. This is so again because (1) states a very *loose* connection between reasons and blameworthiness. Even if we grant the objectivist that we can be completely ignorant of our reasons, there will at least be some possible cases in which we are blameworthy for failing to give the response that is best. When we focus on actions rather than beliefs, such cases will be cases where we either act against our better knowledge of what is best or where we culpably fail to know what is best and do the wrong thing as a result of our ignorance. Analogously, if we are objectivists about reasons for belief—for instance, if we think that we ought believe only what is true or correct to believe rather than what is supported by our evidence –, then we can still argue that we are at least *sometimes* blameworthy because we have a false or incorrect belief. We would be blameworthy in some of the cases where the evidence was accessible to us, and yet did not form the correct doxastic attitude in accord with our evidence. So even if we spell out purely epistemic norms in objectivist terms (e.g., “one ought to believe only what is true”), this does not yet give us an argument against (1). For the accessibility of the evidence does not render the epistemic norm practical, and thus does not compromise the idea that evidence is normative only if we are *sometimes* blameworthy for violating purely epistemic norms.

Finally, one might want to object to (1) by adopting a permissivist epistemology. Permissivism states, roughly, that our total set of evidence permits more than one set of doxastic attitudes to take towards each (or at least some) proposition(s).^15^ According to a permissivist, it could be true that if we have sufficient evidence for a proposition, it is both epistemically permissible to believe it as well as epistemically permissible not to believe it. Such an account might seem to be exactly the conclusion to draw from Harman’s clutter-objection (cf. Sect. 2.1): we are not rationally *obligated* to believe anything that our evidence sufficiently supports, and thus we are not blameworthy for not drawing all implications from our beliefs. But it is always *permissible* for us to believe propositions that are sufficiently supported by our evidence.

However, it is not straightforward how permissivism could pose a problem for (1). The premise states that we can be blameworthy merely for violating a purely epistemic norm if evidence is to provide us with epistemic reasons. To deny this, the permissivist would have to argue that the possibility of epistemic blame is not a necessary condition on the normativity of evidence. They would have to claim that norms of epistemic permissibility provide us with epistemic reasons even if we cannot be blameworthy for violating them.

But this seems false. If something is permitted only under a certain condition, then it is *not* permitted—and thus *prohibited*—if this condition is not fulfilled. That is, that I am permitted to believe that p only if p is sufficiently supported by my evidence implies that I *ought not* to believe that p whenever p is *not* sufficiently supported by my evidence. This is what (EN*) states. Thus, even if we understand the normative force of evidence in terms of permissibility, this will still require us to make sense of the idea that we sometimes *ought* to have certain doxastic attitudes under certain conditions. For instance, when p is insufficiently supported by evidence, we would be required either to suspend judgment about p or to disbelieve p. Purely epistemic norms would then be those norms that require us to either suspend belief about propositions or to disbelieve those propositions that are not sufficiently supported by our evidence (given suitable non-pragmatic background conditions). The normative significance of these norms would still be puzzling if we could not be blameworthy for violating them. Thus, if one reformulates (EN) as a claim about permission rather than obligation, one thereby commits to the equally puzzling epistemic norm (EN*). This won’t make the normative force of reasons for belief any more intelligible if one does not show how one can be blameworthy for violating such purely epistemic norms.

This confirms that (1) is uncontroversial, mainly because it rests on a very loose connection between epistemic reasons and epistemic blameworthiness. The premise can be accepted both by proponents and opponents of the normativity of evidence, by objectivists, and by permissivists alike, and is thus a hinge around which the debate can progress.

It is important to see, however, that (1) would be false if we assume that the relevant sense of “blameworthy” must be a paradigm form of *moral* blame. A person’s epistemic failure does not, by itself, give rise to emotions like resentment, indignation, or guilt. If someone believes that candidate X will win the next elections because the flight of the birds gave them a sign, then our reactive attitudes are not, or not necessarily, of that moral kind. But this does not yet rule out that there could sometimes be a distinct kind of *epistemic* blame appropriate in such cases even when the moral reactions aren’t appropriate. This is also why (2) is not trivial: it claims that there is no such distinctively epistemic kind of blame.

I will now turn to the form of epistemic blame that can legitimately arise due to our epistemic answerability, and to my proposal of how this concept of epistemic blame might help us to understand the normativity of evidence.

## Making the Normativity of Evidence Intelligible: A Proposal

If premise (1) is right—if evidence provides us with epistemic reasons for belief only if we can be blameworthy for violating purely epistemic norms—then a challenge for the normativist about evidence consists in spelling out the nature of the kind of blame that can be appropriate in response to purely epistemic failings, thereby allowing us to see why (2) is false.

There are, of course, other arguments against the normativity of evidence. In the concluding Sect. 5, for instance, I will mention the challenge of making sense of conflicts between purely epistemic norms and practical norms. A full defense of the normativity of evidence would require responding to such challenges as well. Furthermore, a full account of epistemic normativity would have to provide a justification of our epistemic practices—of why we are justified in having a practice of holding each other answerable to purely epistemic norms, responding with reactive attitudes to their violation, and so on. Here I will not provide such a full account. My more modest aim in this paper is rather to make room for the plausibility of a purely epistemic kind of normativity by showing how it reveals itself in our practice of holding each other answerable to epistemic norms. I will return to these issues briefly in the concluding Sect. 5 to clarify what this paper achieves, and what it doesn’t.

To get epistemic blame into focus, I will distinguish between *culpable* and *non-culpable* violations of epistemic norms, and I will discuss briefly how different accounts about responsibility for belief evaluate both cases (Sect. 4.1). The kind of blame that is still appropriate in the non-culpable cases will then give us an idea about how a normativist about evidence could understand epistemic blameworthiness (Sect. 4.2). This will help us to evaluate cases of *trivial* belief in which a purely epistemic norm was violated (Sect. 4.3). The relevant concept of blame will allow us to make sense of the idea that evidence still provides us with reasons to believe when no practical value hinges on what we believe. The proposed view about the normativity of evidence allows us to see that epistemic norms have normative significance even without being hostage to practical value.

### Blameworthiness for Non-culpable Violations of Epistemic Norms

Consider the following distinction between *culpable* and *non-culpable* violations of epistemic norms. There are violations of epistemic norms that we could have reasonably avoided (culpable), and violations which we could not have reasonably avoided (non-culpable). It seems that many deniers of human-induced climate change have plenty of evidence available that should rationally convince them that they are wrong if they would consider their evidence more carefully. Their epistemic norm violation is thus culpable. Like someone who commits a moral wrong for egoistic motives, these people fail to make an effort of will they owe to others by failing to consider their evidence. The epistemic norm violation has severe consequences for other people, and the culpable deniers could have reasonably avoided the violation. Due to this, it can be appropriate to react to them with moral blame, like resentment or indignation: they do not merely violate an epistemic norm, but also a moral norm of belief-management.

Compare the culpable denier of human-induced climate change with the non-culpable denier. The latter person might be someone who grew up in a community where human-induced climate change is denied by everyone even in the face of sufficient evidence. That is, even if members of this group are presented with clear evidence, they remain unconvinced. Consider one of these non-culpable deniers who was trained from an early age on to believe against the evidence when it comes to the topic of climate change. Careful consideration of the evidence is not anything that comes up as a reasonable course of action to this denier, nor can we reasonably expect this of them. Knowing the social background of the denier, we can say that they lost some authority over their beliefs when it comes to human-induced climate change.^16^ This is why resenting them or being indignant does not seem to be appropriate.

Some recent accounts of responsibility for belief would argue that the non-culpable deniers are not blameworthy for their beliefs *in any sense*. They argue that our responsibility for belief is always derivative to our responsibility for actions and omissions prior to that belief by means of which we could have had some reasonable kind of influence or control over the belief (cf. Meylan, 2013, 2017; Peels, 2017)—say, actions of inquiry or investigation, or of actively considering one’s evidence. Consequently, if there was no reasonable course of action for our denier that would have led them to adopt a different doxastic attitude—as we stipulated –, then these accounts imply that our denier is blameless.

Interestingly, given premise (1) of the argument from doxastic blameworthiness, these accounts are committed to denying the normativity of evidence—i.e., they are committed to ANE. For their account implies that a person who holds a blameworthy belief had reasons for actions or omissions by means of which they could have avoided their belief. If so, then a violation of purely epistemic norms does never, by itself, make the person blameworthy. Rather, according to these accounts, blameworthiness presupposes that there were reasons for actions and omissions by means of which the person could have managed their belief. Given (1), ANE follows from this claim.^17^

Other accounts of responsibility for belief would disagree, however. They would argue instead that even the non-culpable deniers might still be *answerable* for their beliefs (cf. Hieronymi, 2006, 2008, 2014; Smith, 2005, 2015). That is, they argue that it might still be intelligible to request of them to *justify* their beliefs by asking them for the evidence they take to bear on whether there is human-induced climate change. In contrast to brute headaches, the deniers’ beliefs still reveal an aspect of their overall epistemic character due to being rationally evaluable. That is, as long as we assume that the non-culpable denier’s beliefs are not wholly unresponsive to reasons, there seems to be a sense in which we could still be justified to react with negative attitudes towards them if they are incapable of providing a satisfying reply to our request for evidence. According to Hieronymi (2009), although the non-culpable denier’s beliefs are not under their indirect *voluntary* control, they might still be conceived of as being under their *evaluative* control: their beliefs might still be active responses to their reason-giving environment (even though they are non-voluntary responses that are irrational), and can thus legitimately give rise to serious forms of criticism or blame.

This is not the place to decide which account of responsibility for beliefs—indirect control accounts or answerability-accounts—are right. Rather than deciding the dispute about what grounds our responsibility for belief, I will instead turn to my proposal about the nature of the blaming-responses that might still be appropriate towards the non-culpable deniers according to the answerability accounts. However, note that this will also pose a problem for indirect control accounts of doxastic responsibility. For if there *is* a kind of distinctively epistemic blame appropriate in the case of the non-culpable deniers, then they are epistemically blameworthy for their beliefs even though they could not have reasonably managed them by exercising indirect control. Most importantly for the present purposes, however, considering the nature of a purely epistemic kind of blame will give us a clue about how to understand the normativity of evidence.

### Epistemic Blame, Relationships, Vices, and Trust

Hieronymi’s (2004, 2019) and Smith’s (2013) approaches to the nature of blame are in line with Scanlon’s (1998, 2008) account. According to this family of accounts of the nature of blame, blaming someone need not mean that one feels emotions like resentment or indignation towards the person. Rather, we might blame someone merely by *modifying our relationship* towards them in a certain way. We might blame a person without feeling any hostility towards them, e.g., by just ceasing to be friends, or by no longer providing special support to the blamee, or by not taking pleasure in their successes, or by not valuing their opinions in the way we did before, or by developing a general sense of distrust towards them. Recently, especially Boult (2020, 2021) has applied these accounts to the epistemic domain. The following sketch of an account of epistemic blame draws on his ideas.^18^

Importantly, not all relationship modifications count as instances of blame. First, one might modify a relationship in a positive way, say, when one becomes so fond of someone that one wants to be closer friends with them; or when a parent finds out that their child committed a crime and in response to this cares even *more* about them (Smith, 2013, 137). Secondly, relationship modifications can happen without negative judgment about another person—as when people who live in different places just drift apart. Scanlonian approaches to blame thus owe us an account of what makes a *negative* relationship modification an instance of blame.^19^

Negative relationship modifications count as instances of blame only if they are responses to the person’s vice. I do not count as blaming my friend by judging and treating them as unreliable, and by modifying my expectations accordingly, if I am aware that their unreliability is due to factors that do not stem from their faulty character. Such factors might include their newborn child that makes them spontaneously cancel on me, or their depression that is the cause for their unreliability. Such factors do not give me a reason not to trust them, but merely a reason not to *rely* on them. By contrast, it might be legitimate to blame a friend if their unreliability indicates that they do not care about the friendship as much as one can reasonably expect of them as a friend. In this case, they are not fully honest about their attitude towards the friendship. Reducing one’s trust in them, and thus modifying one’s relationship with them negatively in response to their vice of dishonesty, can be legitimate.^20^

Negative relationship modifications in response to vices can plausibly count as blaming responses because they are only legitimate towards responsible beings. This is because we can only have the specific kind of relationship that is presupposed by these reactions with fully responsible beings. Neither computer nor children or animals can display vices that give rise to the negative reactions described above. Their misbehavior can only give rise to impoverished analogues of these reactions. For instance, I might “not trust” a dog in the sense that I suspect that they will bite me. The dog’s behavior might be unreliable, but it won’t give me a reason to blame the dog, since the dog’s behavior does not manifest a vice (on the assumption that dogs cannot have full-blown vices like fully responsible beings). This indicates that negative relationship modifications in response to vices presuppose a subject’s responsibility for their character and attitudes. At the same time, the appropriateness of these reactions does not presuppose that the subject could have managed their character or attitudes: these reactions merely presuppose an underlying vice, independently of its origin in voluntary conduct. Since these reactions presuppose responsibility, their potential “coolness” does not, *pace* Wallace (2011), count against them as genuine blaming-reactions (cf. Boult, 2020).^21^

As David Owens puts it, after discussing the epistemic vice of gullibility: when I display a vice indicating a flaw in my character, then “I cannot be trusted to think and feel as I ought” (2000, 124). The normativity of these “oughts” is revealed, according to view I propose, by the fact that violating them impairs our relationship to others in specific ways so that it becomes appropriate to negatively modify one’s relationship—e.g., by reducing one’s presumption of epistemic trust. This impairment exists even if the person had no opportunity to manage their vice: as long as the epistemic vices are still genuine *vices* (rather than pathologies), non-culpable violations of epistemic norms that reveal a person’s epistemic vice can impair our epistemic relationships, and thus give rise to suspension of epistemic trust.

If we allow for a broad concept of blame in terms of impaired relationships, then we might be able to make room for something like purely epistemic blameworthiness. In an initial attempt, we might state that if we are blameworthy *morally* as soon as our relationship to our *moral* community is impaired, then we are blameworthy *epistemically* as soon as our relationship to our *epistemic* community is impaired. This impairment might matter in specific ways for how we should relate to one another: whether we believe the other person, whether we provide them with information, and whether we engage with them in rational discourse.

One problem with this initial formulation is that one’s moral or epistemic community can be epistemically or morally flawed, and thus one might end up impairing one’s relationship with them by being morally or epistemically virtuous.^22^ Boult’s (2020) formulation of the position avoids this problem: one is blameworthy epistemically only if one falls short of the *normative ideal* of an epistemic relationship—or, in my preferred terminology, only if one displays an epistemic vice. The epistemically virtuous person does not fall short of this ideal even within an epistemically flawed community. Thus, members of the community won’t have a reason to reduce their epistemic trust in the virtuous person. By appealing to the normative ideal of an epistemic relationship, we can explain why being dogmatic or gullible, engaging in wishful thinking, or being biased can make one epistemically blameworthy even in epistemic communities that socially reward such vices. For all these vices are, as Boult puts it, problematic ways of exercising one’s epistemic agency that make one fall short of the normative ideal and thus warrant suspension of one’s presumption of epistemic trust.^23^

### Blameworthiness for Non-culpable and for Trivial Violations of Epistemic Norms

Let us apply this sketch of an account of epistemic blame to our two relevant cases: *non-culpable* violations and *trivial* violations of epistemic norms. In both cases, it seems as if passionate forms of moral blame, like resentment or indignation, are no longer appropriate. Importantly, however, reducing our presumption of epistemic trust for failing to live up to the normative ideal of an epistemic relationship might still be appropriate. This will allow us to see that we already take epistemic norms to be normatively significant: we are already committedly involved in epistemic sociality.^24^ Our actual practice of holding each other answerable to purely epistemic norms presupposes their normative significance insofar as we express this significance in our reactive attitudes towards norm violators.

The non-culpable deniers of climate change might still be manifesting an epistemic vice, a defect in character, which we might label “epistemic irrationality”.^25^ Note first that their epistemic irrationality is *attributable* to them in the sense that it is part of their overall outlook on the world, rather than just an occasional lapse which we could excuse. It is thus a genuine vice. Secondly, our non-culpable norm violators are still *answerable* for their beliefs insofar as it is intelligible to request their *evidence* for their beliefs. For we conceived of the case in such a way that their disbelief is still rationally evaluable rather than pathological: we assumed that their belief is irrational in the sense that they are aware, on some level, of the evidence against their belief, but they still fail to respond correctly to their overall evidence due to epistemic vices like dogmatism, gullibility, or a tendency for wishful thinking. Our epistemic blame directed at the non-culpable deniers is based on our judgment that they cannot give a satisfying answer to our request for evidence, even though the evidence is readily available to them. As a result, we have a reason to suspend our presupposition of epistemic trust towards them.^26^

What is the verdict, according to this account of epistemic blame, about our blameworthiness for trivial belief that is insufficiently supported by one’s evidence? The normativist about evidence has two strategies available. Both strategies can be combined.

First, normativists could argue that even violations of epistemic norms in trivial matters might indicate a general flaw in the epistemic character of a person. As Boult puts it when evaluating trivial cases, “[s]o long as I modify my intentions and expectations towards them, in a way made fitting by the judgment (however implicit) that they’ve impaired the general epistemic relationship, then I count as epistemically blaming them” (Boult, 2020, 9). That is, if your friend tends to believe celebrity gossip that they read in a magazine they know to be unreliable, this might give you a (*pro tanto* or *prima facie*) reason to suspend epistemic trust in them. Presumably, this could mean that you should suspend your trust in some situations when it comes to matters of importance, because you now have some evidence that their epistemic character is flawed.

Secondly, the normativist can just grant that violations of epistemic norms do not *always* make it appropriate to suspend trust. For they need not argue that such violations *always* make one epistemically blameworthy. In order to disprove premise (2), it is enough to show that we are *sometimes* blameworthy in virtue of the fact that we violated a purely epistemic norm. More generally, violating a reason-providing norm need not amount to displaying a criticizable vice, and thus need not amount to blameworthiness. Compare the idea that someone’s morally wrong action is not necessarily blameworthy. We all act wrong from time to time, and we all violate epistemic norms from time to time. We can usually *excuse* each other for occasional lapses and do not regard these lapses as having any significant consequences for our interpersonal relationships. Yet moral wrongs and violations of epistemic norms are lapses nevertheless—i.e., they are violations of norms that provide us with reasons for compliance.

Seeing that reducing epistemic trust is an appropriate negative response to an epistemic vice and that it marks the impairment of an epistemic relationship provides us with a plausible starting point for understanding the significance of epistemic normativity. It allows us to meet the challenge for the normativity of evidence presented in this paper by rejecting premise (2). This challenge claims that the absence of a distinctively epistemic kind of blame rules out the normativity of evidence. I have proposed that we can meet this challenge by appealing to recent accounts of doxastic responsibility as answerability and to recent accounts of epistemic blame. The former accounts show us that non-culpable beliefs might still be blameworthy. The latter accounts provide us with an idea about what this blameworthiness could consist in, and how it could sometimes extend also to cases of trivial belief. By building on Boult’s account, I have suggested that epistemic blame consists in marking impaired epistemic relationships by reducing epistemic trust in response to a person’s epistemic vice.

## Conclusion and Outlook

The dispute about the normativity of evidence is a currently lively discussion within epistemology.^27^ There is no need right now to settle the dispute once and for all. This paper has contributed two ideas towards bringing the debate forward:

- That a central challenge in defending the normativity of evidence consists in spelling out a notion of a distinctively *epistemic* kind of blame.
- That appealing to epistemic blame as marking an impaired epistemic relationship by suspending epistemic trust in response to epistemic vices is a promising way for defending the distinctive normativity of evidence in the sense required by this challenge.

Together, both claims shift the dialectical burden towards ANE. Proponents of ANE argue that purely epistemic norms do not provide us with reasons to believe. But if epistemic blame marks the violation of purely epistemic norms with reason-providing force, then proponents of ANE must say that there is no such thing as a distinctively epistemic kind of blame. However, recent approaches on doxastic responsibility as answerability (Hieronymi, Smith), as well as recent works that spell out the nature of a distinctively epistemic kind blame or criticism (Boult, Brown, Kauppinen), call this into doubt. As a result, proponents of ANE need to engage with these theories: they have to show why the appropriateness of the blaming-reactions that these theories spell out does not imply that a norm with reason-providing force was violated; or else argue that these reactions are not appropriate in response to violations of purely epistemic norms. However, I have suggested in Sect. 4 that violations of epistemic norms in non-culpable and trivial cases can well deserve suspension of epistemic trust if they are manifestations of epistemic vice. The presented analysis of these cases thus calls into doubt ANE by revealing a purely epistemic kind of blame that might be appropriate in these cases—a blame that reflects the normative significance we attach to purely epistemic norms.

However, this does not yet provide us with a full account of epistemic normativity. I will now briefly explain what I think such an account requires, at a minimum. This will reveal the restrictions of the present inquiry. At the same time, it illustrates how the approach presented in this paper might be fruitfully developed to a fuller account of epistemic reasons.

The first requirement for a full account of epistemic normativity is that it must allow us to meet other challenges for the normativity of evidence. For instance, how do normativists about evidence deal with cases in which complying with an epistemic norm causes practical disvalue—for instance, cases in which others will suffer harm unless I make myself violate an epistemic norm? The proposed view about epistemic blame might help us to make sense of the traditional verdict that I *ought epistemically* to comply with the epistemic norm even though I *ought practically* to bring myself not to comply with it. Proponents of ANE will argue that the first “ought” cannot by normatively authoritative in any interesting sense. The proposed view, by contrast, allows us to say that the first epistemic “ought” has still a kind of normative authority insofar as the normative significance of this “ought” is expressed in the fact that members of one’s epistemic community might be justified in modifying their trusting attitude towards me if I do not comply with it. That is, even if I bring myself to violate an epistemic norm for good practical reasons, I might end up not being trustworthy epistemically due to the resulting ill-based belief. This will hold at least in cases where my resulting ill-based belief reflects an epistemic vice. However, the discussion about such cases is currently very alive, and this paper has no ambitions meeting this and further possible challenges.^28^

Secondly, this paper did not provide an account of the *source* of epistemic normativity. My appeal to our actual practice reveals that we treat each other as answerable to epistemic norms, and thus that we attach normative significance to these norms. But this does not justify our commitment to the overall epistemic practice. Indeed, the view proposed here is even compatible with a *pragmatic* foundation of purely epistemic norms: maybe we are justified to engage in our epistemic practice because it is practically valuable to be subject to epistemic norms (cf. Owens, 2017). Combined with such a pragmatic justification of our overall practice, normativists about evidence could maintain that *within* this practice, all reasons for belief are provided by our evidence, and that pragmatic considerations are only relevant if we wish to externally justify our adherence to this purely evidential kind of normativity.^29^ This might be an important element in a complete error-theory about pragmatist-instrumentalist intuitions concerning reasons for belief.

## Footnotes

1 In their terminology, epistemic norms are just not *authoritatively* normative. However, according to their view, also the rules of chess count as (non-authoritatively) normative. In my terminology, norms of chess are *not* normative in the sense that they do not provide us with reasons for compliance. I follow Kiesewetter (2017, 3–4) in this use of “normative”. In Maguire’s/Wood’s terminology, I am interested in *authoritative* norms and reasons. While I use the term “norm” as including also non-normative rules or standards, I use “reasons” exclusively in a normative sense. I do not think there are non-normative reasons (except for explanatory ones). So my question is: “Do epistemic norms provide us with reasons?” Cf. Hofmann (2020) on some unpacking of the idea that some norms “provide” us with reasons—on which I cannot elaborate within the scope of this paper.

2 For recent accounts with similar implications, cf. Bondy (2018), Cowie (2014), Mantel (2019), McCormick (2015, 2020), Papineau (2013). Of these, only McCormick identifies explicitly as a pragmatist (like Rinard). Instrumentalists argue that they can avoid a commitment to pragmatism—i.e., to the existence of practical reasons for belief per se—by saying that it is always *evidence* that motivates our beliefs (rather than practical reasons). Instrumentalists merely claim that evidence *gains its normative authority* from practical considerations (cf. Cowie, 2014, 4004–5; Steglich-Petersen & Skipper, 2019, 11). I am not here interested in subtle differences between the versions of pragmatism and instrumentalism I consider. I am only interested in their implication of ANE. (However, cf. note 10 below on how Steglich-Petersen/Skipper’s version of instrumentalism might collapse into pragmatism.).

3 One might doubt that I do not believe the disjunctive proposition. For if I was asked whether I believe it, and I understand the content of the proposition, I will reply that I do believe it. However, even if one thinks that we believe all those disjunctive propositions, one will agree that we do not believe all the implications of our current belief-stock, like certain mathematical or logical implications that are just too hard to figure out. That we do not believe those implications does not make us blameworthy or criticizable in any sense. Furthermore, we *can* imagine a case where I fail to believe such weird disjunctive propositions. Why on earth, we might ask, should any reasonable person care about this so much as to regard me as blameworthy for not believing them?

4 For the sake of brevity, I use the notion “blameworthiness” in what follows to cover both the notion of blame and other forms of personal criticism (cf. Kiesewetter 2017, ch. 2 on the notion of *personal* criticism as contrasted with, say, criticism of a knife for not being sharp). One might think that “blameworthiness” is essentially a moral notion. As I will argue in Sect. 4, epistemic blame is a *sui generis* kind of blame. This might warrant the label “criticism” instead of “blame” for epistemic blame. However, I take this to be a mere terminological issue.

5 Kiesewetter is not concerned with background conditions on (EN*). But his view might naturally be extended to (EN*) in the way described. As argued at the end of the last section, a background condition on (EN*) is as important as a background condition on (EN) to make it intelligible that these norms have reason-providing force.

6 Cf., e.g., Rinard (2015, 220), Steglich-Petersen (2011, 23) for this verdict about structurally analogous cases.

7 It is important to note that Steglich-Petersen does not think that this background condition is necessary for (EN*) to express an epistemic requirement. However, as I have argued in Sect. 2.1, he thereby ignores that (EN*) is faced with very similar challenges as (EN): Why give up a lot of beneficial beliefs that are insufficiently supported by one’s evidence? And why blame or criticize anyone for believing something that is insufficiently supported by their evidence if it doesn’t matter at all whether they believe it? It seems that Steglich-Petersen’s instrumentalist framework commits him to a background condition not only for (EN), but also to (EN*): without assuming pragmatic background conditions, both norms seem questionable as genuine normative requirements.

8 Note that the reason for forming a belief about p cannot be itself an epistemic reason for belief, because epistemic reasons favor believing a specific proposition—they favor believing that p or not believing that p. Steglich-Petersen’s reason to form a (true) belief about p, by contrast, does not favor believing a specific proposition. It merely favors having a true belief about a matter, whatever this belief turns out to be. Only practical reasons can favor forming a belief about a matter without favoring a specific proposition.

9 In later works, Steglich-Petersen accepts that the reason to form a belief about p must in fact be a reason to form a *true* belief about p (see esp. the formulations of the norms of belief in Steglich-Petersen & Skipper, 2019, 2020).

10 One might wish to interpret Steglich-Petersen’s view without committing to the idea that the practical reason is always a reason for an action of managing one’s beliefs (causing, maintaining, etc.). However, as pointed out in note 8 above, the “reason to form a belief about whether p” cannot be epistemic. It must thus be practical. If the practical reason does not favor an action, it must favor the state of believing itself. This would, however, commit Steglich-Petersen to pragmatism about reasons for belief. Whether this reason favors actions of managing beliefs or rather beliefs themselves, the condition renders the requirement practical rather than purely epistemic.

11 Cf. note 4 on my use of “blameworthy”.

12 Cf. note 2 for proponents.

13 A recent account of epistemic blame which is clearly motivated in this way is Kauppinen (2018). On the side of the proponents of ANE, McCormick (2020) argues that all blame for belief is ultimately prudential or moral. She *therefore* rejects that there is a normative domain of the purely epistemic. Thus, she explicitly reaches ANE by accepting (1) and (2) (cf. McCormick, 2020, 30). The problem with McCormick’s argument is that she does not consider the epistemic forms of blame that have been spelled out by Boult (2020, 2021), Brown (2020), and Kauppinen (2018), and to which I will appeal in Sect. 4.2. She rather thinks of blame mainly in moral terms.

14 For some discussion and an argument for subjectivism that builds on the notion of *praise*worthiness, cf. Lord (2018, ch. 8). See also Kiesewetter (2017, ch. 8) for a good overview of the debate and another case for subjectivism.

15 The denial of permissivism is often discussed as the *Uniqueness Thesis* (as introduced by Feldman, 2007). On epistemic permissivism and some of its problems, cf. White (2005).

16 In the terminology employed by Fischer and Ravizza (1998) and McCormick (2015), we might say that they lost their *ownership* over the relevant belief-forming mechanisms.

17 That these accounts are committed to a denial of the normativity of evidence might give rise to a strong argument against them. As far as I know, this implication has not been noticed before. I suppose that this is because the relationship between reasons and blameworthiness (or responsibility) is underexplored.

18 Cf. his cited works for a detailed defense. Other accounts of epistemic blame have been worked out by Kauppinen (2018), who also presents an account of epistemic criticism as a form of distrust, and by Brown (2020), who also shares the spirit of Boult and Kauppinen in that she regards epistemic blame as being neither a mere negative evaluation nor a kind of strong reactive emotion (like resentment). However, Brown builds on Sher’s (2006, 2009) account of blame in order to spell out an epistemic kind of blame. Neither Kauppinen nor Brown put relationship modifications at center stage. Cf. Boult (2021) for a detailed discussion and critique of Brown’s account.

19 In response to Smith’s case mentioned in the paragraph above, Boult (cf. 2021, 17) argues that genuine blaming reactions are those that are based on the judgment that the person is blameworthy. However, a problem with this proposal is that it does not tell us what our judgments about blameworthiness are based on. My proposal (that I explain in the next paragraph) avoids this problem: our judgments of blameworthiness are responses to the blamee’s vice. Furthermore, it seems that the parent in Smith’s case regards their child as blameworthy without blaming them. I think Smith’s case can be met simply by restricting blaming responses to *negative* relationship modifications. Maybe Smith’s (2013) proposal that blaming responses are expressions of one’s protest is compatible with my proposal that they are based on epistemic vice—maybe epistemic blame expresses protest against epistemic vices. However, cf. Boult (2021) for some skepticism about the applicability of Smith’s account to the epistemic domain.

20 Cf. Smith’s (2005, 242) case from George Eliot’s *Scenes of Clerical Life* (1858), where Captain Wybrow fails to notice that Miss Assher never takes jelly, which, according to Smith, “suggests to Miss Assher that she does not yet occupy a distinctive place in his overall emotional and evaluative outlook” (2005, 243). Assher might legitimately modify her expectations towards Wybrow, and thus modify the relationship negatively, in response to Wybrow’s vice of not caring about the relationship as much as he should. According to the proposed account, this would count as an instance of blame even if it does not involve emotions of resentment or indignation.

21 An anonymous referee objected that negative relationship modifications might merely count as ways of *holding responsible*, but not of ways of *blaming*. This raises the question of how full-blown moral blaming responses like resentment and indignation relate to the “softer” Scanlonian negative reactive attitudes. For my purposes here, it is sufficient to note that if we hold a person responsible in the Scanlonian sense, this can provide the basis for a positive or a negative relationship modification in response to virtue or vice. For instance, we might want to be closer friends because of the person’s virtues, or we might reduce our involvement with the person due to their vices. I take it to be of secondary interest whether we *call* these reactions “praise” and “blame”. At the very least, they seem to be positive and negative ways of holding responsible. This is sufficient for these reactions to reveal the normative significance of a norm that we mark as violated by reacting in these ways. For defenses of such reactions as genuine *blaming* responses, cf. the recent works that argue that these reactions count as blame because they go hand in hand with, or consist in, a kind of motivation—a desire that the blamee had not “believed badly” (Brown, 2020), a protest against the blamee’s action or attitude (Smith, 2013) or just generally the motivation to change one’s relationship with them by modifying one’s expectations and intentions (Boult 2021).

22 I am grateful to an anonymous referee for making me aware of this problem.

23 I take it that Boult does not use “epistemic agency” as referring to indirect voluntary control over beliefs. Plausibly, one can be dogmatic, gullible, a wishful thinker or biased even if this was not under one’s indirect voluntary control. Rather, beliefs—including irrational ones—are often involuntary responses to one’s environment (cf. Strawson, 2003). Boult’s notion of epistemic agency is more plausibly understood in terms of Hieronymi’s (2009) notion of evaluative control.

24 I owe this idea to Matthew Chrisman.

25 On rationality as a virtue, cf. Wedgwood (2017). Although I do not wish to make any substantial claims about rationality here, note that the idea I present in this paragraph fits well with the argument that irrationality (which can be non-culpable) is criticizable and must therefore be connected to, or even spelled out in terms of, normative reasons (cf., e.g., Kauppinen, 2019, 3; Kiesewetter, 2017, chapter 2; Schmidt, forthcoming; Way, 2009, 1).

26 Importantly, things would be rather different if the non-culpable deniers were not in possession of sufficient evidence for human-induced climate change. For instance, we might conceive of a community that managed to shut off any evidence from the outside world, and that worked on discrediting science within their community over decades. In this case, disbelieve in human-induced climate change might turn out to be rational for them, given their misleading epistemic perspective. In this case, we might not *rely* on their judgments. But we then won’t count as blaming them—their unfortunate circumstances would excuse most individuals within the community. They would not count as epistemically vicious, because they are epistemically rational to a normal degree.

27 Cf. also the recent discussion about the normativity of epistemic reasons. Cf. Kiesewetter (forthcoming) for an overview over this debate and defense of the normativity of epistemic reasons. According to Kiesewetter, some instrumentalists or pragmatists are best read as making room for a notion of a non-normative epistemic reason, rather than as denying the existence of epistemic reasons altogether. My terminology here does not allow us to distinguish between these two positions, because I used ‘epistemic reason’ exclusively in a normative sense.

28 Heil (1992) gives a helpful impression of the two opposing intuitions in such cases: (a) there are two incommensurable or incomparable senses of “ought” *versus* (b) only the practical ought matters. For recent defenses and elaborations along the lines of (b), cf. Howard (2020), Maguire and Woods (2020), Meylan (2020), Reisner (2008), Rinard (2017), Steglich-Petersen and Skipper (2019). For verdicts along the lines of (a), cf., next to Heil (1992), Berker (2018) Feldman (2000, 680–681), Kelly (2003, 619), Pojman (1993).

29 Cf. this view with Dennett’s parallel view about the justification of punishment: while a practice of punishment can be externally justified, according to Dennett, only by consequential considerations (deterrence, re-socialization, etc.), our particular judgments about who is to be punished are justified by desert-based considerations (who deserves to be punished, whom it is unfair to punish, etc.) that are internal to this practice (cf. Dennett & Caruso, 2021, 119–127). Analogously, our beliefs might be justifiable only by purely evidential considerations while our overall practice must be justified on pragmatic grounds.
